# Supplementary material for: SlATG8f modulates tomato thermotolerance and fruit quality, correlating with changes in autophagy and heat shock-related genes
Source: PLoS One. 2026 Jan 9;21(1):e0334005. doi: 10.1371/journal.pone.0334005 (PMC12788623; doi:10.1371/journal.pone.0334005)
Supplement: S1 Table — (PDF) [file pone.0334005.s001.pdf]

Table 1： Real-time fluorescence quantitative primer information

Gene ID	Gene	Primer Sequences(5'→3')
Solyc01g068060	*SlATG8a*-F	CGTGAAGCAAAGAAATGGGGAAGAC
	SlATG8a-R	ACCGGCACACGATCAGGATATTTG
Solyc02g080590	*SlATG8b*-F	TTATGTGGTGCGGAAGAGGATCAAG
	SlATG8b-R	AGAGAAGCTGTTGGAGGCAAAGTG
Solyc03g031650	*SlATG8c*-F	TCCGAAAGAGGATCAATCTCAGTGC
	SlATG8c-R	CAGACATCAGAGCAGCAGTTGGAG
Solyc03g078400	*Actin*-F	GAAATAGCATAAGATGGCAGACG
	Actin-R	ATACCCACCATCACACCAGTAT
Solyc02g067760	*MYB21*-F	TTGCCTGGAAGAACGGATAACG
	MYB21-R	TTCCTTGTCCATTCATGTTCTCTCC
Solyc03g121740	*MYB26*-F	CAACAAACGCGGCTATCAAA
	MYB26-R	GAGCACTCTTGACCACCATG
Solyc08g062960	*HsfA2*-F	TGGTTATTATGGGATGGAGGAGGAG
	HsfA2-R	GTACTCTGCTGTTGCTGCCTAAG
Solyc09g009100	*HsfA3*-F	AGTCTCCTTGTCTATGTTGCTTGG
	HsfA3-R	AAGATGTCTATGGGTGTGGTTTGG
Solyc01g009200	*HSP20*-F	ATTAAGGCGTCTATGGAGAATGGAG
	HSP20-R	ATGGACTTGACATCAGGCTTCTTC
Solyc03g082400	*HSP21*-F	ACAATGCGGCAGATGATAGACAC
	HSP21-R	CCCTGTTCCTGATGCTCTGTTTC
Solyc04g009320	*HSP70*-F	CACTGCTGCTTCATTGGCTTATG
	HSP70-R	CTCCATCTCCAACCTCAAGAACTG
Solyc06g036290	*HSP90*-F	GCAGAGAACAAGGAGGACTACAAC
	HSP90-R	AGGAGGTCAGCCAACTTAGCC
Solyc08g007400	*SlATG8e*-F	TTGACGGTAGGGCAGTTTGTGTATG
	SlATG8e-R	TGGAAGACATGATTGCACCTGTTGG
Solyc08g078820	*SlATG8f*-F	ACAATGTGCTACCGCCAACAGG
	SlATG8f-R	GGTAACATAGAGGAAGCCGTCATCG

Solyc10g006270	*SlATG8g*-F	TTGGTTCCTGCTGATCTAACTGTGG
	SlATG8g-R	TGCTCCTCATAAATGGCGGACATC
Solyc07g064680	*SlATG8d*-F	GCTGCCATGATGTCTGCCATTTATG
	SlATG8d-R	GAAGGTATTCTCGCCACTGTAGGTC
