# Supplementary material for: SlATG8f modulates tomato thermotolerance and fruit quality, correlating with changes in autophagy and heat shock-related genes
Source: PLoS One. 2026 Jan 9;21(1):e0334005. doi: 10.1371/journal.pone.0334005 (PMC12788623; doi:10.1371/journal.pone.0334005)
Supplement: S2 Table — (PDF) [file pone.0334005.s002.pdf]

Table 2：qRT-PCR reaction system and reaction program.

Ingredients	Volume of use
Template	1 μl
Primer F	2 μl
Primer R	2 μl
2X Taq PCR Master Mix II	25 μl
Sterilized ddH_2O	20 μl

Reaction Temperature	Response Time	Cycle Number
94℃	5 min	1
94℃	30 s	
50℃	45 s	30
72℃	1 min	
72℃	10 min	1
16℃	30 min	1
