# Supplementary material for: SlATG8f modulates tomato thermotolerance and fruit quality, correlating with changes in autophagy and heat shock-related genes
Source: PLoS One. 2026 Jan 9;21(1):e0334005. doi: 10.1371/journal.pone.0334005 (PMC12788623; doi:10.1371/journal.pone.0334005)
Supplement: Attachment — Submitted filename: Response to Reviewers docx..docx [file pone.0334005.s004.docx]

**Manuscript ID:**PONE-D-25-51450R1

**Title:** *SlATG8f* modulates tomato thermotolerance and fruit quality, correlating with changes in autophage and heat shock-related genes.

**Author name:**Qunmei Cheng;Wen Xu

***Reply to the comments***

**Dear Editors and Reviewers:**

We express our gratitude to the editors and reviewers for their time, constructive feedback, and insightful comments on our manuscript. Your input has significantly enhanced the quality of our research. All raised points were thoughtfully addressed, and necessary revisions have been highlighted in red in the revised manuscript. Detailed responses to each reviewer's feedback are presented below.

**Reply to the comments of Editors:**

***Comments:* (1)**Please ensure that your manuscript meets PLOS ONE's style requirements, including those for file naming. The PLOS ONE style templates can be found at https://journals.plos.org/plosone/s/file?id=wjVg/PLOSOne_formatting_sample_main_body.pdf and https://journals.plos.org/plosone/s/file?id=ba62/PLOSOne_formatting_sample_title_authors_affiliations

***Reply:*** We express our gratitude to the editorial office for highlighting the style requirements. After a meticulous review of the PLOS ONE style templates provided, we have diligently revi-sed our manuscript to ensure strict adherence to all formatting guidelines, encompassing file na-ming conventions, manuscript structure, and reference formatting. We are confident that the ma-nuscript now fully complies with the journal's style requirements and appreciate your professio-nal guidance in preparing our work for publication.

We eagerly anticipate your further feedback.

***Comments:* (2)**We note that the grant information you provided in the“ Funding Information” and “Financial Disclosure” sections do not match.When you resubmit, please ensure that you provide the correct grant numbers for the awards you received for your study in the“Funding Information” section.

***Reply:*** We are grateful to the editorial office for highlighting the discrepancy in the grant info-rmation. Following a thorough review, we have rectified the funding details in both the “Fund-ing Information” and “Financial Disclosure” sections to ensure complete alignment, encompassi-ng all grant numbers and award specifics. Your meticulousness in bringing this matter to our attention is acknowledged, and we have confirmed the coherence of all funding information in the revised manuscript.

We eagerly anticipate your ongoing guidance throughout the review process.

***Comments:* (3)**Thank you for stating the following financial disclosure:“The authors are grateful to the National and Local Vegetable Engineering Center (Guizhou) and the Laboratory of the Department of Horticulture (Agricultural College of Guizhou University) for their support to t-his project.”

- Please state what role the funders took in the study.  If the funders had no role, please state: "The funders had no role in study design, data collection and analysis, decision to publish, or preparation of the manuscript."
- If this statement is not correct you must amend it as needed.
- Please include this amended Role of Funder statement in your cover letter; we will change the online submission form on your behalf.

***Reply:*** We are grateful to the editorial office for their assistance in updating the financial discl-osure statement. The funding details have been rectified, and the necessary acknowledgment ofthe funders' role has been incorporated. The revised funding statement is as follows:

This work was supported by the National Natural Science Foundation of China (32260754, 31760594) and the Platform construction project of the Engineering Research Center for Pr-otected Vegetable Crops in Higher Learning Institutions of Guizhou Province (Qian Jiao Ji [2022] No. 040).The funding agency provided the experimental site and technical support.

We have incorporated the revised statement into our cover letter as per your request. We appreciate your help in ensuring that our manuscript complies with the journal's guidelines and eagerly anticipate your additional feedback on our submission.

***Comments:* (4)** Please include captions for your Supporting Information files at the end of your manuscript, and update any in-text citations to match accordingly. Please see our Supporting Information guidelines for more information: http://journals.plos.org/plosone/s/supporting-information.

***Reply:*** We express our gratitude to the editorial office for prompting us regarding the supporting information requirements. Detailed captions for all supporting information files have been inc-luded at the end of the manuscript, and relevant in-text citations have been updated accordingly. These modifications adhere completely to PLOS ONE's Supporting Information guidelines. We value your guidance in assisting us to adhere to the journal's formatting standards and anticipate your additional feedback on our submission.

***Comments:* (5)** If the reviewer comments include a recommendation to cite specific previously published works, please review and evaluate these publications to determine whether they are r-elevant and should be cited. There is no requirement to cite these works unless the editor hasindicated otherwise.

***Reply:*** We thank the editorial office for the reminder about evaluating potential references. Aft-er a thorough review of all reviewer comments, we have found no specific recommendations f-or citing additional publications. If the editor believes certain references are crucial for providi-ng context to our work, we are open to including them.

We value your guidance and await further instructions on our manuscript.

***Comments:* (6)**Please review your reference list to ensure that it is complete and correct. If yo-u have cited papers that have been retracted, please include the rationale for doing so in the manuscript text, or remove these references and replace them with relevant current references. Any changes to the reference list should be mentioned in the rebuttal letter that accompanies y-our revised manuscript.

If you need to cite a retracted article, indicate the article’s retracted status in the References list and also include a citation and full reference for the retraction notice.

***Reply:*** Our sincere appreciation to the editorial office for the reminder to verify the reference list. Upon careful review, we have confirmed the absence of any references to retracted papersin our manuscript. Furthermore, we have added new relevant references in the revised version,highlighted in red for easy identification. The reference list is now comprehensive and precise. We are grateful for your valuable guidance and await your feedback on our revised man-uscript.

**Reply to the comments of Reviewer #1**

***Comments:* (1)**The title “*SlATG8f* enhances tomato thermotolerance and fruit quality via autoph-agy and HS pathways” assumes the mechanism without proving it. The data shows a correlatio-n, not causation, so the title must be changed to accurately reflect the data. Recommend revise-d title: *SlATG8f* modulates tomato thermotolerance and fruit quality, correlating with changes i-n autophage and heat shock-related genes.

***Reply:*** Our sincere appreciation goes to the reviewer for the insightful comment and for reco-

mending a more accurate title. We acknowledge that our data illustrate a correlation rather thandefinitive causation. As a result, we have decided to adopt the reviewer’s proposed title verbati-m: “*SlATG8f* modulates tomato thermotolerance and fruit quality, correlating with variations in

autophagy and heat shock-related genes”. **Please refer to the revised page 1 for details.**

***Comments:* (2)** The current structure is a disjointed list. Reorganize into clear subheadings to

improve readability.

***Reply:*** We appreciate the reviewer’s valuable suggestion regarding the need for a clearer structure, as it enhances readability. Accordingly, we have meticulously restructured the “Materials and Methods” section by incorporating descriptive subheadings to establish a more cohesive and logical progression. **Please refer to the revised page 4-8 for details.**

- Plant Materials and Growth Conditions
- Generation of Transgentic Tomato Lines
- Heat Stress Treatment and Sample Collection
- Phenotypic Assessment of Fruit Development
- Analysis of Fruit Quality Parameters
- Measurement of Antioxidant Enzyme Activities and Malondialdehyde (MDA) Content
- RNA Extraction and Gene Expression Analysis (qRT-PCR)
- Statistical Analysis

We are grateful for your valuable guidance and await your feedback on our revised manuscript.

***Comments:* (3)** Ensure consistent formatting for gene names. Some are Italicized,others are not.

***Reply:*** Our sincere thanks to the reviewer for the detailed observation. We regret the error in

gene name formatting and have now meticulously reviewed the entire manuscript to ensure theconsistent italicization of all gene names (e.g., *SlATG8f, ATG8, SlHSP90*) throughout the text, f-igures, and figure legends, in compliance with standard scientific nomenclature. Protein names continue to be presented in nonitalicized text.

***Comments:*（4**Please unify “*SlATG8f*-overexpressing (OE)” and “*SlATG8f OE*”by using the corre-ct form “*SlATG8f OE* plants”.

***Reply:*** We thank the reviewer for this helpful comment. We have now carefully revised the en-tire manuscript to consistently use the unified term “*SlATG8f OE plants”* (or “*SlATG8f-OE fru-its”* where contextually appropriate) to describe the transgenic lines, as suggested.

We believe this change improves the clarity and consistency of the text.

***Comments:* (5)**“Random sampling was performed” is vague, describe how the samples were selected and pooled if applicable.

***Reply:*** We appreciate the reviewer’s insightful comment and acknowledge the need for a moreprecise description of the sampling method. Accordingly, we have enhanced the “Heat Stress T-reatment and Sample Collection” subsection in the “Materials and Methods” section to offer ac-omprehensive overview of our sampling procedure.explicitly outlines the random selection of fr-uits from various positi-ons on multiple plants for each biological replicate, and elucidates theprocess of pooling tissu-e samples for analysis. **Please refer to the revised page 4-5 for detai-ls.**

***Comments:* (6)** “conventional soilless substrate” is not specific. The number of fruits sampled per stage, per plant, per treatment is not stated. A concise summary is required.

***Reply:*** We appreciate the reviewer's constructive suggestions and have accordingly revised the manuscript to incorporate the specific details as advised.

- In the “Plant Materials and Growth Conditions” section, the soilless substrate composition is detailed as follows: Seedlings were grown in a soilless substrate consisting of peat, vermiculite, and perlite in a volume ratio of 2:1:1.
- In the “Heat Stress Treatment and Sample Collection” subsection, the sampling strategy is clearly outlined: “For each of the four developmental stages, nine fruits (three from each of three randomly selected plants) were collected per genotype per treatment to serve as biological replicates for further analyses.”

We are grateful for your valuable guidance and await your feedback on our revised manuscript.

***Comments:* (7)**Correct the numerous typographical and spacing errors.

***Reply:*** We express our gratitude to the reviewer for their meticulous observation. Following thi-s feedback, we conducted a thorough proofreading of the manuscript, rectifying all typographic-al and spacing errors to align with the journal's formatting guidelines.

We are grateful for your valuable guidance and await your feedback on our revised manus-cript.

**Reply to the comments of Reviewer #2**

***Comments:* (1)**Title: the manuscript does not analyze the thermotolerance, I suggest remove thermotolerance from the title. Furthermore, HS should write the full name.

***Reply:*** We thank the reviewer for bringing up these important points. In response to the suggestion regarding the term “thermotolerance,” we have carefully considered it. In the field of plant stress physiology, the capacity of plants to maintain functionality under high-temperature stress is viewed as an active response, demonstrating thermotolerance throughout their growth and development[1,2]. However, to accurately represent our correlational data，we have replaced the term “enhances”with “modulates” and presented the finding as a correlation. We believe that the revised title now effectively reflects our findings without excessive interpretation. Concerning the abbreviation “HS” we concur and have expanded it in the revised title to "heat shock.

We are grateful for your valuable guidance and await your feedback on our revised manus-cript.

***Comments:* (2)**Key words: “High temperature stress” changed to“ high-temperature stress”.

***Reply:*** We sincerely thank the reviewer for this careful correction. We have revised the key words from “High temperature stress” to “high-temperature stress” as suggested.

***Comments:* (3)**Introduction: the role of autophagy in fruit development and quality need descripts.

***Reply:*** We appreciate the valuable suggestion and acknowledge the importance of including background information on the role of autophagy in fruit development and quality in the introduction. Consequently, we have incorporated a new paragraph in the Introduction section to address this aspect. **Please refer to the revised page 3 for details.**

We thank you once again for this insightful recommendation.

***Comments:* (4)**Many gene names should be in Italic, please check and modified. For instance, *ATG* in page 2.

***Reply:*** Thank you for highlighting the importance of gene name formatting for standardization and professionalism in the paper. We apologize for any formatting errors in the original text. F-ollowing your guidance, we have meticulously reviewed and corrected the entire manuscript inaccordance with the journal's author guidelines. All gene names, including members of the *AT-G* gene family (*e.g., ATG5, ATG7, ATG8a*), the core gene *SlATG8f* studied here, and related g-enes, have been consistently italicized. This adjustment has been applied to the abstract, introd-uction, results, discussion, and figure legends throughout the document.

We appreciate your thorough review, which has significantly enhanced the quality of our s-ubmission.

***Comments:* (5)**Abbreviations are defined upon their first appearance; subsequent occurrences require only the abbreviation itself, without repeating the definition.

***Reply:*** We appreciate the reviewer for highlighting this oversight and apologize for any inconsi-stency in abbreviation usage. Following a thorough review, we have ensured that each abbrevi-ation is defined only upon its initial mention in the abstract, main text, and figure legends. S-ubsequent references now utilize the abbreviation alone. For instance, “heat stress (HS)”is defin-ed once, and “quantitative reverse transcription PCR (qRT-PCR)” is not reiterated in the Meth-ods section.

We are grateful for your valuable guidance.

***Comments:* (6)**“L” in the Latin name for tomato should not be italic.

***Reply:*** We express our gratitude to the reviewer for highlighting the significant technical detailconcerning the taxonomic nomenclature. The Latin name for tomato has been rectified througho-ut the manuscript to adhere to the correct format, “*Solanum lycopersicum* L”. Furthermore, wehave meticulously reviewed all scientific names in the text to ensure consistency with this stan-dard. We value your thoroughness in enhancing the accuracy of our manuscript.

***Comments:* (7)**Delete “novel” in page 3.

***Reply:*** The reviewer’s specific recommendation to remove the term “novel”as instructed on pag-e 3, is duly acknowledged. A comprehensive review of the manuscript has been carried out t-ensure the removal of similar subjective assertions. Emphasizing the importance of letting the f-indings speak for themselves,

we are grateful for this adjustment to enhance the objectivity of the language.

***Comments:* (8)**M & M: F2 should be subscript of 2.

**Reply:** We sincerely thank the reviewer for highlighting this formatting error. We have now co-rrected “F2” to “F₂” in the “Plant Materials and Growth Conditions”section of the Materials and Methods. now consistently use the correct subscript format. We appreciate your attention to detail in helping us improve the manuscript's presentation.

***Comments:*（9）**Agrobacterium and its Latin name should be italic.

**Reply:**We are grateful to the reviewer for identifying the oversight in the microbial nomenclature formatting. The genus and species name “*Agrobacterium tumefaciens*” has been appropriatelyitalicized in the “Generation of Transgenic Tomato Lines” section. We have confirmed that all microbial names in the manuscript now comply with this standard.

Your assistance in maintaining the accuracy of our scientific terminology is valued.

***Comments:* (10)**Results: the conclusion in the last paragraph in page 6 is not consistent with the results of Fig. 2. The results for fruit set were not description.

**Reply:** We appreciate the reviewer for highlighting the inconsistency and lack of detail regarding the fruit set results. After a thorough re-analysis of the data presented in Fig. 2, we have revised the corresponding description in the Results section. The revised text provided below addresses the inconsistency and offers a clear and precise interpretation of Fig. 2.

Revised Text inManuscript: In parallel, WT plants demonstrated a higher fruit set rate than *SlATG8f OE* plants under normal conditions, but heat stress notably decreased fruit set in WT plants. Conversely, *SlATG8f OE* plants maintained a consistent fruit set rate under stress conditions. Although no significant difference in fruit set was noted between WT and *SlATG8f OE* lines under high temperature, the transgenic plants effectively mitigated the heat-induced decline in fruit set.

We extend our gratitude to the reviewer for their meticulous review and constructive feedback, which has enhanced the clarity and accuracy of our results presentation.

***Comments:* (11)**Figures: the images require reformatting. Consider grouping the results for the same metric across four periods into a single row.

**Reply:** We sincerely thank the reviewer for this valuable suggestion regarding figure organizati-on. We have now reformatted all relevant figures (e.g., Fig 1, Fig 2, Fig 3, and Fig 4,Fig 5,Fig 6) by grouping data for the same metric across the four developmental stages into single ro-ws,

We express our deepest gratitude to the reviewers and editorial team for their exceptional guidance and support during the revision process. The insightful comments and constructive suggestions provided have significantly enhanced the quality, clarity, and scientific rigor of our manuscript. The thorough and thoughtful feedback has not only strengthened the paper but also enriched our understanding of the subject. It has been a privilege to collaborate with such a dedicated and professional team. We appreciate your time, expertise, and contribution to making this a rewarding scholarly experience.

Yours sincerely，

Wen Xu

16th November,2025

**Reference**

1．Huo L, Sun X, Guo Z, Jia X, Che R, Sun Y, et al. *MdATG18a* Overexpression Improves Basal Thermotolerance in Transgenic Apple by Decreasing Damage to Chloroplasts. Hortic Res. 2020;7:21. pmid: **32140230.**
2．Cappetta E, Andolfo G, Guadagno A, Di MatteoA, Barone A, Frusciante, et al. Tomato Genomic Prediction for Good Performance under High-Temperature and Identification of LociInvolved in Thermotolerance Response. Hortic Res. 2021; 8:1–16. pmid: **34593775.**
