# Supplementary material for: SlATG8f modulates tomato thermotolerance and fruit quality, correlating with changes in autophagy and heat shock-related genes
Source: PLoS One. 2026 Jan 9;21(1):e0334005. doi: 10.1371/journal.pone.0334005 (PMC12788623; doi:10.1371/journal.pone.0334005)
Supplement: Attachment — Submitted filename: Response to Reviewers.docx [file pone.0334005.s005.docx]

**Manuscript ID:**PONE-D-25-51450R1

**Title:** *SlATG8f* modulates tomato thermotolerance and fruit quality, correlating with changes in autophagy and heat shock-related genes.

**Author name:**Qunmei Cheng;Wen Xu

***Reply to the comments***

**Dear Editors:**

We express our gratitude to the editorial office for the invaluable guidance provided during this second round of revision. All the points raised in your latest editorial comments have been thoroughly addressed, as outlined below. Your ongoing support is greatly appreciated, and we eagerly anticipate your final evaluation of our revised manuscript.

**Reply to the comments of Editors:**

***Comments:* (1)** If the reviewer comments include a recommendation to cite specific previously published works, please review and evaluate these publications to determine whether they are relevant and should be cited. There is no requirement to cite these works unless the editor has indicated otherwise.

***Reply:***We express our gratitude to the editorial office for the reminder regarding potential citati-on recommendations. Upon thorough re-evaluation of all reviewer comments, we confirm that no specific suggestions were made to cite additional publications. If the editor deems any parti-cular references necessary for the context of our study, we are willing to incorporate them as advised. Your guidance is highly valued, and we eagerly await your further evaluation of our revised manuscript.

***Comments:* (2)**Please review your reference list to ensure that it is complete and correct. If yo-u have cited papers that have been retracted, please include the rationale for doing so in the manuscript text, or remove these references and replace them with relevant current references. Any changes to the reference list should be mentioned in the rebuttal letter that accompanies y-our revised manuscript. If you need to cite a retracted article, indicate the article’s retracted st-atus in the References list and also include a citation and full reference for the retraction noti-ce.

***Reply:*** We express our gratitude to the editorial office for the reminder to ensure the com-pleteness and accuracy of the reference list. All references have been meticulously reviewed a-nd their formatting updated to adhere fully to the journal’s style guidelines. There are no citat-ions of retracted articles in this manuscript:

In response to previous review feedback, we have enhanced the manuscript by incorporating the following reference updates, all highlighted in red in the tracked-changes version:

Added a supporting reference in the Introduction to substantiate the statement: “While auto-phagy in crops has been extensively studied in contexts such as leaf senescence, seed development, and vascular formation”.

Included several pertinent references in the newly integrated section discussing the role of autophagy in fruit development and quality.

After a comprehensive assessment, I have determined that there is no need to cite a retrac-ted article. Therefore, I have not indicated the retraction status of the article in the reference list, nor have I cited the retraction notice separately or included the complete reference.

We affirm that the reference list is now precise, comprehensive, and free of retracted publ-ications. We value your guidance and anticipate your evaluation of our revised manuscript.

***Comments:* (3)**The edits you made to the manuscript are satisfactory. However, I request that you correct the word autophage in the title of your manuscript to autophagy. Please make thiscorrection and resubm.

***Reply:*** We are grateful to the editors for their favorable assessment of our revisions and for identifying the typographical error in the manuscript title. The correction from “autophage” to “autophagy” has been made as per their suggestion. We value the reviewer's meticulous review and await their final evaluation of our manuscript.

We express our deepest gratitude to the The manuscript has been revised to fulfill all editorial requirements. We appreciate your guidance throughout the review process. We are confident that these revisions have enhanced the paper and trust that it now meets the publication standards of PLOS ONE.

Yours sincerely，

Wen Xu

December 14, 2025
